# Sociodemographic analysis of the psychiatric domiciliary hospitalization (DH) program at Hospital General Universitario of Ciudad Real (HGUCR), Spain

**DOI:** 10.1192/j.eurpsy.2025.812

**Published:** 2025-08-26

**Authors:** M. E. Sánchez-Escalonilla, E. Segura, A. Muñoz, C. Herranz, M. Viaña, I. Diéguez, Á. Esquembre

**Affiliations:** 1 Hospital Universitario La Paz, Madrid; 2 Hospital Universitario de Ciudad Real, CR, Spain

## Abstract

**Introduction:**

Domiciliary hospitalization emerged in the late 20th century as a new mental health intervention, designed to providing care of patients with mental disorders at home. This approach offers benefits to hospitals, patients and their surroinding support systems (Megías, F. et al. EVES 2004; 16: 11-107). Although numerous international studies have evaluated the quality and advantages of domiciliary hospitalization, there is a paucity of research in Spain.

**Objectives:**

To describe and compare the sociodemographic characteristics of psychiatric patients admitted to the Short-term Hospitalization Unit (UHB) at HGUCR with those of patients admitted to DH Unit from January 1 to December 31, 2019; and to compare this results with the statistics found in other similar studies.

**Methods:**

This study is a descriptive observational analysis of 281 patient admissions to psychiatric hospitalization units (UHB or DH) in 2019 at HGUCR. The variables analyzed include the type of hospitalization, age, sex, marital status, type of cohabitation and employment status. SPSS was used as a statistical analysis tool. A literature review was carried out, using PubMed to identify comparable national and international studies.

**Results:**

The mean age of patients was 44±15 years, with no significant differences between patients admitted to UHB and those admitted to DH unit, consistent with findings from other studies. The percentage of men and women is similar, with a majority of singles (40%) or married/in partnership (38%) compared to those who were separated or widowed. Regarding types of cohabitation, 37% of patients lived with their own family, 35% with their family of origin and 18% lived alone, with no significant differences between the two types of hospitalization. As regards employment status, the largest group (27%) was inactive, followed by 18% who were incapacitated and 17% of active workers. Significant differences were found in employment status: there were more active people in DH and more incapacitated individuals in UHB. However, we found that in other studies from Germany, there were significantly more unemployed people in DH (Bechdolf et al. FDNP 2011; 79 (1): 26-31). This can be explained by the exlusion criteria for DH at HGUCR, which include patients with severe social problems and multiple or decompensated organic comorbidity. Therefore, it is consistent that most of DH patients were in relatively good health and capable of working actively before admission.

**Conclusions:**

No significant differences between DH and UHB were observed in most variables. Where differences were observed, they could be explained by the differing exclusion criteria between the two types of hospitalization. Our results are similar to those reported in other studies.

**Disclosure of Interest:**

None Declared

